# Glutathione and glutamate in schizophrenia: a 7T MRS study

**DOI:** 10.1038/s41380-018-0104-7

**Published:** 2018-06-22

**Authors:** Jyothika Kumar, Elizabeth B. Liddle, Carolina C. Fernandes, Lena Palaniyappan, Emma L. Hall, Siân E. Robson, Molly Simmonite, Jan Fiesal, Mohammad Z. Katshu, Ayaz Qureshi, Michael Skelton, Nikolaos G. Christodoulou, Matthew J. Brookes, Peter G. Morris, Peter F. Liddle

**Affiliations:** 1 Division of Psychiatry and Applied Psychology, University of Nottingham, Nottingham, UK; 2 Sir Peter Mansfield Imaging Centre, University of Nottingham, Nottingham, UK; 3 Departments of Psychiatry, Medical Biophysics and Neuroscience, Western University, London, ON Canada; 4 Lawson Research, Brain and Mind & Robarts Research Institutes, London, ON Canada; 5 Healthcare Improvement Scotland, Gyle Square, Edinburgh, UK; 6 Department of Psychology, University of Michigan, Ann Arbor, MI USA; 7 South Staffordshire and Shropshire Healthcare NHS Foundation Trust, Stafford, UK; 8 Nottinghamshire Healthcare NHS Foundation Trust, Nottingham, UK; 9 Greater Manchester West Mental Health NHS Foundation Trust, Manchester, UK; 10 Derbyshire Healthcare NHS Foundation Trust, Derby, UK

**Keywords:** Schizophrenia, Neuroscience

## Abstract

In schizophrenia, abnormal neural metabolite concentrations may arise from cortical damage following neuroinflammatory processes implicated in acute episodes. Inflammation is associated with increased glutamate, whereas the antioxidant glutathione may protect against inflammation-induced oxidative stress. We hypothesized that patients with stable schizophrenia would exhibit a reduction in glutathione, glutamate, and/or glutamine in the cerebral cortex, consistent with a post-inflammatory response, and that this reduction would be most marked in patients with “residual schizophrenia”, in whom an early stage with positive psychotic symptoms has progressed to a late stage characterized by long-term negative symptoms and impairments. We recruited 28 patients with stable schizophrenia and 45 healthy participants matched for age, gender, and parental socio-economic status. We measured glutathione, glutamate and glutamine concentrations in the anterior cingulate cortex (ACC), left insula, and visual cortex using 7T proton magnetic resonance spectroscopy (MRS). Glutathione and glutamate were significantly correlated in all three voxels. Glutamine concentrations across the three voxels were significantly correlated with each other. Principal components analysis (PCA) produced three clear components: an ACC glutathione–glutamate component; an insula-visual glutathione–glutamate component; and a glutamine component. Patients with stable schizophrenia had significantly lower scores on the ACC glutathione–glutamate component, an effect almost entirely leveraged by the sub-group of patients with residual schizophrenia. All three metabolite concentration values in the ACC were significantly reduced in this group. These findings are consistent with the hypothesis that excitotoxicity during the acute phase of illness leads to reduced glutathione and glutamate in the residual phase of the illness.

## Introduction

Schizophrenia is a serious, episodic, and persisting illness, with a characteristic time course in which acute episodes, characterized by positive psychotic symptoms such as delusions and hallucinations, are followed by a chronic phase in which negative symptoms and disabling cognitive and social impairments tend to be prominent. There is evidence for three potentially related pathophysiological processes consistent with the characteristic time course of illness.

Firstly, abnormally high levels of presynaptic striatal dopamine occur in at least some patients, and are associated with positive symptoms [1]. Treatment with dopamine antagonists tends to alleviate these positive symptoms. Secondly, the brain’s major excitatory neurotransmitter, glutamate, is implicated [2]. One proposal is that hypofunction of NMDA receptors (NMDAR) located on inhibitory GABA inter-neurons leads to reduced activity of these inter-neurons, resulting in increased pyramidal glutamatergic neurotransmission and the diverse symptoms occurring in acute psychosis [3]. However, sustained glutamatergic over-activity might lead to excitotoxic damage resulting in residual negative symptoms, cognitive dysfunction, and occupational and social impairment [4]. Glutamatergic abnormalities in schizophrenia have been found in brain tissue studies [5]; genetic association studies [6]; and studies investigating the role of glutamatergic agonists to combat NMDAR hypofunction and reduce symptoms [7].

Thirdly, and potentially closely related to the glutamatergic hypothesis, is the theory of oxidative stress [8]. Oxidative stress can result either from the excessive production of reactive oxygen species, which might be anticipated at times of glutamatergic over-activity, or from a reduction in oxidative defences, and can damage cell structures. Brain cells are particularly susceptible, owing to their low antioxidant defences and high oxidative metabolic activity. Glutathione, the brain’s major intracellular antioxidant, thus has a vital role to play in maintaining brain cell health. Evidence for a role for oxidative stress in schizophrenia includes studies reporting reduced glutathione blood levels [9], reduced post-mortem glutathione concentrations [10, 11], and genetic evidence implicating schizophrenia-related polymorphisms in genes involved in glutathione synthesis [12].

Animal studies indicate a close relationship between glutamate and glutathione. Persson et al. [13] showed that glutathione synthesis was directly related to microglial glutamate uptake and the release of glutamate metabolites. Barger et al. showed that depletion in glutathione levels due to oxidative stress is related to microglial glutamate release [14]. In a review of the role of glutathione in protection against neuronal death, Bains et al. [15] present evidence that glutamatergic transmission activates biochemical pathways that generate free radicals and/or lower defence against damage by free radicals. Conversely, free radicals can increase the concentration of glutamate in the synaptic cleft by the release of glutamate or blockade of its re-uptake. In particular, oxidative stress reduces the uptake of glutamate in astrocytes, a key step in the recycling of glutamate [16]. This potentially creates a vicious cycle leading to damage by free radicals unless the glutamatergic transmission is associated with increase in glutathione.

Other evidence implicates NMDA receptors in the interaction between glutathione and glutamate. In a recent review article, Hardingham and Do [17] presented evidence indicating that NMDAR hypofunction and oxidative stress may be reciprocally linked. For instance, glutathione enhances NMDAR responses whereas its depletion results in NMDAR hypofunction [18]. Furthermore, NMDAR hypofunction can also lead to oxidative damage [19]. Baxter et al. [20] demonstrated that synaptic activity, mediated via NMDA receptors, boosts the synthesis and utilization of glutathione, thereby adjusting antioxidant capacity to meet the elevated needs of active neurons. These studies indicate that in an equilibrium state there is likely to be a close coupling between glutamate and glutathione.

Magnetic resonance spectroscopy (MRS) allows biochemical concentrations of glutathione, glutamate, and other metabolites to be measured non-invasively in vivo. One MRS study found reduced glutathione in the medial prefrontal cortex (mPFC) in schizophrenia patients compared with healthy controls [21]. Another recent study reported reduced glutathione only in schizophrenia patients carrying a risk variant of the gene coding for the rate-limiting enzyme in glutathione synthesis [22]. Four other studies reported no statistically significant differences in glutathione levels in the posterior medial frontal cortex [23], mPFC [24] or anterior cingulate cortex (ACC) [25, 26]. However, two out of the four found a trend toward reduced glutathione in schizophrenia [24, 25] and in one, lowered levels of glutathione were associated with negative symptoms [24]. On balance, these studies of predominantly well-established cases of schizophrenia indicate a tendency toward reduced glutathione. In contrast, a study of first-episode cases reported elevated glutathione in medial temporal lobes [27] raising the possibility that glutathione abnormalities might vary with phase of illness. However, it should be noted that this study used a lenient threshold for the inclusion of glutathione metabolite fits.

Because glutamate released during neurotransmission is recycled via glutamine in glial cells, glutamine may index glutamate neurotransmission. However, glutamate and glutamine each participate in several other distinct cell processes and hence may not be closely coupled [28]. MRS studies of glutamate and glutamine concentrations in schizophrenia have been inconsistent, with some studies reporting an increase, others reporting a decrease or no abnormality ([29–35], for reviews see [36, 37]). One meta-analysis found that glutamate is reduced and glutamine is increased in the ACC in patients with schizophrenia, and both decrease more markedly with age in patients [38]. A more recent meta-analysis found higher medial-frontal Glx (glutamate + glutamine) levels in high-risk subjects [39]. Three recent studies used ultra-high field strength (7T) MRS, as its greater spectral resolution and signal-to-noise ratio help to distinguish between these two metabolites. Two studies investigated the ACC/mPFC and one of these studies found increased glutamine/glutamate ratio [40] while the other found no significant abnormalities in glutamate levels in schizophrenia [41]. A third study found reduced glutamate in the occipital cortex in schizophrenia patients compared to healthy controls [42]. These studies provide some evidence of elevated glutamine levels in ACC/mPFC, especially in the early phase of illness. Overall, these studies indicate that glutamate may be unchanged or reduced, especially in more chronic cases, depending on the persistence of symptoms or other factors associated with long-term illness [29–37].

We reasoned that if decreases in glutathione and glutamate in the stable phase are a consequence of damage arising in a preceding acute phase, a sample of stable patients would be more likely to demonstrate a reduction in these neurochemicals than a more heterogeneous sample. We also predicted that any reduction in these neurochemicals would be most marked in cases of residual schizophrenia. ICD-10 defines residual schizophrenia as a chronic stage of the illness, in which there has been a progression from an early stage with positive psychotic symptoms to a later stage characterized by long-term negative symptoms and impairments, but reduced frequency and severity of positive symptoms [43]. Although the DSM-IV [44] does not explicitly define residual schizophrenia, it describes as typical a course of the illness in which positive symptoms diminish while negative symptoms persist. If residual schizophrenia reflects neural damage due to oxidative stress, reduced levels of glutamate and glutathione should be most marked in patients with residual schizophrenia.

As much of the MRS evidence in schizophrenia relates to the ACC, this was our primary region of interest. Insular cortex together with ACC forms the Salience Network, which is hypothesized to play an important role in schizophrenia [45]. Moreover, evidence indicates progressive structural changes in the insula early in the illness [46]. We therefore placed a voxel in the left insula. While much of the focus has been on the frontal cortex, it is possible that abnormalities in glutamatergic neurotransmission are widespread and include the visual cortex [47]. Hence, we chose the visual cortex as our third region of interest.

Since animal studies have demonstrated a close link between glutathione and glutamate uptake into both microglia and astrocytes, and NMDA receptor function, we also predicted that glutathione and glutamate levels in the brain would be correlated across all subjects. Although our primary hypothesis concerns glutathione and glutamate, the underlying hypothesis is that in the residual state there is diminished neural metabolic integrity arising from a toxic process in the acute phase of illness [4]. We might therefore predict that other metabolites would also exhibit reductions correlated with reductions in glutathione and glutamate. We therefore examined the correlations between glutathione, glutamate, and other metabolites reflecting cellular metabolic processes.

## Methods

### Participants

Patients aged 18–55 with a diagnosis of schizophrenia or schizoaffective disorder were referred to the study by community-based mental healthcare teams in Nottinghamshire, Derbyshire and Lincolnshire, England (for inclusion and exclusion criteria, see SA1 (Supplement).

All patients were in a stable phase of illness, defined as a change of no more than 10 points in their Social and Occupational Functioning Assessment Scale (SOFAS) score (defined in DSM-IV [44]) between assessment 6 weeks prior to and immediately prior to study participation. Patients with a documented history of prominent positive symptoms of schizophrenia but currently exhibiting no substantial positive symptoms were deemed to satisfy ICD-10 criteria for residual schizophrenia if they exhibited appreciable negative symptoms, and/or impaired occupational and/or social function. Operational criteria are specified formally in SA2.

Most patients were receiving psychotropic medication. The median defined daily dose (DDD) [48] was calculated separately for antipsychotics, mood stabilizers including lithium, and antidepressants. No patient had had a change in any of these medications 6 weeks prior to participating in this study.

Healthy participants with no personal or family history of psychotic disorders were recruited from the local community via posters, and matched group-wise to the patient group for age, gender, and parental socio-economic status (SA3). Exclusion criteria were as for the patients.

All participants were assessed on scanning day for handedness, social and occupational functioning, and IQ. A clinical interview by a trained team member using a standardized symptom assessment was video-recorded (SA4).

This study was approved by the National Research Ethics Committee. All participants gave informed consent and received an inconvenience allowance.

### Image acquisition

**Fig. 1:**
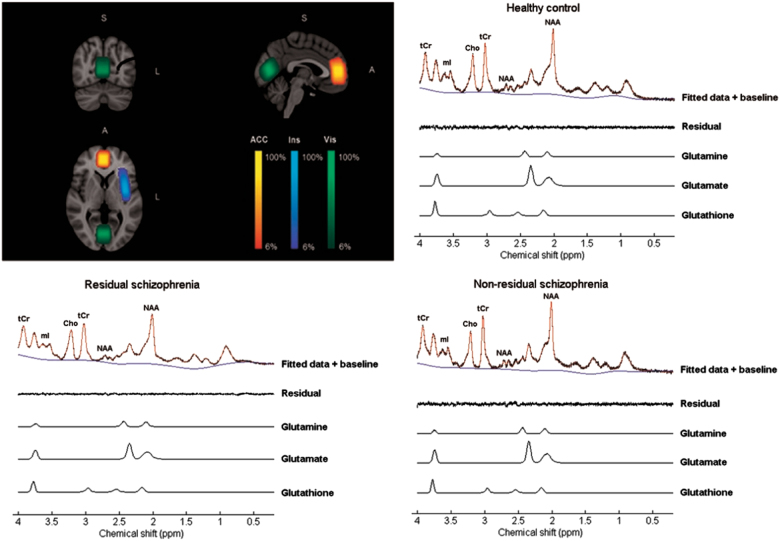
Average voxel placements (ACC anterior cingulate cortex, Ins left insula, Vis visual cortex) across all subjects overlaid on an MNI brain; illustrative ^1^H spectra with baseline and residuals from a voxel located in the ACC of subjects from all three groups (healthy controls, residual schizophrenia, and non-residual schizophrenia) and corresponding fits from LCModel for glutamine (Gln), glutamate (Glu), and glutathione (GSH)

Scans were conducted at the Sir Peter Mansfield Imaging Centre, University of Nottingham, using a 7 Tesla Philips Achieva scanner (Philips Medical Systems, Best, The Netherlands), a volume transmit head coil, and a 32-channel receive head coil. MRS data were collected using a ^1^H-MRS single voxel short TE STEAM (STimulated Echo Acquisition Mode) sequence (TE/TM/TR = 17/17/2000 ms) with eight phase cycle steps, 4096 samples, and a 4 kHz bandwidth. Two-hundred and eighty-eight spectra were collected using the Multiply Optimized Insensitive Suppression Train (MOIST) technique for water suppression. Two spectra were collected without water suppression in order to correct for absolute concentrations using water referencing. A B_0_ field map was acquired and parcellated shimming was used to enhance B_0_ homogeneity [49]. The primary voxel of interest (VOI) (20 × 18 × 25mm^3^) was placed in the ACC; a second VOI (40 × 12 × 18mm^3^) was placed in the left insula; and third VOI (20 × 22 × 20mm^3^) in the visual cortex. See Fig. 1 for a sample spectrum and voxel placements. An anatomical T1 MPRAGE image (TE/TR = 3.4/7.3 ms) was acquired for each subject (1 mm isotropic resolution, 256 × 256 × 180 matrix, flip angle 8°) to aid placement of the VOIs and for co-registration.

### Data processing

For details of the procedure for estimating the metabolite concentrations in the three voxels see SA5. Briefly, the anatomical image was segmented into grey matter, white matter, and cerebrospinal fluid. MRS data acquired independently from the 32 channels were combined using an optimized coil combination method [50]. Metabolite concentrations were estimated using LCModel [51]. Data exceeding a threshold of SD = 20% for the Cramer-Rao Lower Bound estimate of the precision of the quantification were excluded from the analysis. Metabolite concentrations were normalized to the concentration of water calculated from unsuppressed water spectra, since variations in water content within the sample are expected to be much smaller than the variations in the metabolite level [52]. Tissue volume fractions determined from the segmented images were used to correct metabolite concentrations for partial volume effects and relaxation attenuation in each voxel [53]. Tissue composition and MRS data quality measures are reported in ST1–ST3.

### Statistical analyses

As Pearson correlation coefficients between the nine metabolite concentration values (three metabolites in three voxels) were substantial even after adjustment for potential confounders, we used principal component analysis (PCA) to identify latent metabolite variables, and extracted scores for each component (see Results and SA7 for details). The PCA was weighted by group size; missing values were replaced by the mean. Statistical analyses were performed using SPSS 22 (IBM).

To determine, firstly, whether mean metabolite component scores in patients with stable schizophrenia differ from mean scores in healthy controls, and secondly, whether this difference is significantly leveraged by the sub-group of patients with residual schizophrenia, we regressed each component score in turn on two participant variables using a hierarchical regression model. In Block 1, we entered a binary dummy variable, “Stable Schizophrenia”, coded 1 for a patient with schizophrenia and 0 otherwise. In Block 2, we entered an additional binary dummy variable, “Residual Schizophrenia”, coded 1 for a patient meeting criteria for residual schizophrenia, and 0 otherwise.

To facilitate comparison with other studies, we also used independent samples *t*-tests to test for differences in metabolite concentrations between controls and (1) all patients, and (2) patients with residual schizophrenia. We also calculated Pearson correlations between glutathione, glutamate, glutamine, creatine, myo-inositol, and *n*-acetyl aspartate, and between metabolite PCA component scores and age; and antipsychotic DDD.

## Results

### Participants

Data from 45 healthy participants and 28 patients with schizophrenia were included in the analysis. Thirteen of the patients met criteria for residual schizophrenia (see SA6 for details of exclusions and a discussion relating to our sample size, clinical and demographic features of the sample are reported in ST4).

### LCModel results

Means for glutathione, glutamate, and glutamine concentrations (estimated by LCModel and corrected for tissue fraction) in each of the three voxels are given in Table 1 for healthy controls, all patients with stable schizophrenia and in the sub-group of patients with residual schizophrenia (for other metabolites see ST5).

**Table 1:** Mean glutathione (GSH), glutamate (Glu), and glutamine (Gln) concentrations (measured in millimolar (mM) and corrected for tissue fraction) in healthy controls (HC); all patients with stable schizophrenia (All Stable SZ), and in patients with residual schizophrenia (Residual SZ) each of the three regions—anterior cingulate cortex (ACC), left insula, and visual cortex.

		HC		All Stable SZ		Residual SZ		HC vs All Stable SZ					HC vs Residual SZ				
		N	M (SD)	N	M (SD)	N	M (SD)	Diff.	*t*	df	*p*	Hedge’s *g* (LCL, UCL)	Diff.	*t*	df	*p*	Hedge’s *g* (LCL, UCL)
	*ACC*																
	Glutathione	45	1.75 (0.31)	27	1.55 (0.26)	12	1.49 (0.23)	−0.21	2.890	70	0.005	−0.7 (−1.19, −0.21)	−0.26	2.731	55	0.008	−0.87 (−1.53, −0.22)
	Glutamate	45	6.21 (0.81)	27	6.01 (0.66)	12	5.68 (0.73)	−0.20	1.092	70	0.278	−0.26 (−0.74, 0.22)	−0.53	2.061	55	0.044	−0.66 (−1.31, −0.01)
	Glutamine	42	1.66 (0.31)	27	1.48 (0.36)	12	1.40 (0.30)	−0.18	2.241	67	0.028	−0.55 (−1.04, −0.05)	−0.26	2.581	52	0.013	−0.83 (−1.49, −0.17)
	*Insula*																
	Glutathione	45	1.72 (0.20)	27	1.68 (0.26)	12	1.70 (0.24)	−0.04	0.695	70	0.489	−0.17 (−0.65, 0.31)	−0.02	0.276	55	0.784	−0.09 (−0.73, 0.55)
	Glutamate	45	6.44 (0.51)	27	6.39 (0.68)	12	6.56 (0.6)	-0.05	0.364	70	0.717	−0.09 (−0.56, 0.39)	0.11	−0.659	55	0.513	0.21 (−0.43, 0.85)
	Glutamine	44	1.57 (0.28)	26	1.59 (0.30)	11	1.56 (0.24)	0.02	0.325	68	0.746	0.08 (−0.41, 0.56)	−0.01	0.067	53	0.947	−0.02 (−0.68, 0.64)
	*Visual*																
	Glutathione	45	1.50 (0.17)	26	1.47 (0.20)	11	1.46 (0.24)	−0.03	0.739	69	0.462	−0.18 (−0.66, 0.30)	−0.05	0.738	54	0.464	−0.24 (−0.91, 0.42)
	Glutamate	45	5.40 (0.51)	26	5.30 (0.51)	11	5.37 (0.55)	−0.11	0.845	69	0.401	−0.21 (−0.69, 0.28)	−0.03	0.181	54	0.857	−0.06 (−0.72, 0.6)
	Glutamine	44	1.2 (0.23)	26	1.22 (0.25)	11	1.19 (0.30)	−0.07	1.213	68	0.229	−0.30 (−0.78, 0.19)	−0.11	1.320	53	0.192	−0.44 (−1.1, 0.23)

Differences between HC and All Stable SZ means, and between HC and Residual SZ means are given (HC mean−Patient mean) together with *t*-test results and effect size estimates (Hedge’s *g*, with 95% confidence limits). Assumptions of normality and homoscedasticity were satisfied in all comparisons

### Correlations and confounds

**Fig. 2:**
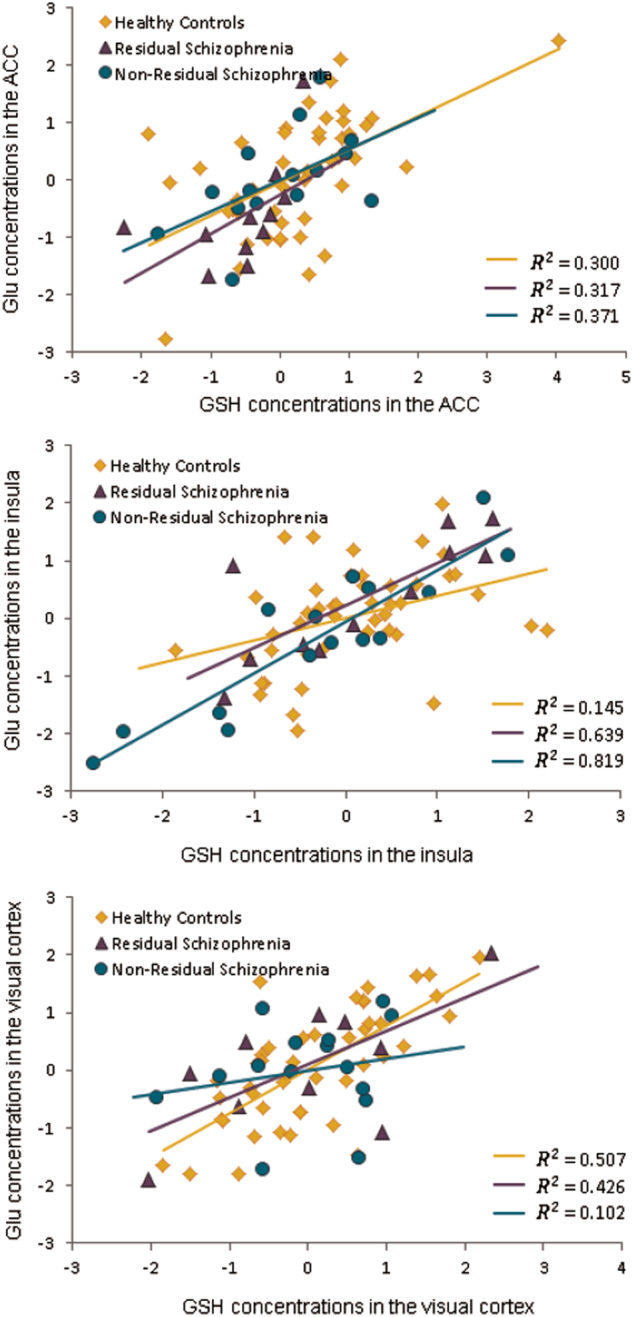
Correlation between glutathione (GSH) and glutamate (Glu) concentrations in the three regions (anterior cingulate cortex (ACC), visual cortex, and insula) in the three groups (healthy controls, patients with residual schizophrenia, and patients with non-residual schizophrenia). Values represent standardized residuals after covarying for gender in the ACC and insula; and gender and spectral line-width in the visual cortex. Note: For the correlation between GSH and Glu in the ACC in healthy controls and in the insula in patients with residual schizophrenia, data did not satisfy the assumption of multivariate normality. Therefore, bootstrapped bias-corrected accelerated 95% confidence intervals were computed (10,000 samples), and the correlations remained significant

As metabolite concentrations tended to be systematically higher in men than in women, we adjusted the values for gender (see SA7 for details). We found significant bivariate Pearson correlations between adjusted glutathione and glutamate in all three voxels (*p* < 0.05) (Fig. 2). Glutamine levels were significantly correlated across all three voxels, with *r*-values ranging from 0.28 to 0.36 (*p* < 0.05). The pattern of correlations between metabolites in the patient and control groups examined separately was similar to that in the combined group.

The PCA of the nine adjusted metabolite concentration values (glutathione, glutamate, and glutamine in each of the three voxels, ACC, Insula, and Visual) resulted in three components with an eigenvalue greater than 1. Component loadings after varimax rotation are given in Table 2. The first component (accounting for 28% of the variance) loaded positively on glutathione and glutamate measures in the visual and insula voxels. The second component (18.7% of the variance) loaded positively on glutathione and glutamate in the ACC and the third component (18% of the variance) loaded positively on glutamine measures in all three voxels. Separate PCA in patient and control groups identified three similar components with the same metabolite loadings exceeding 0.5 on corresponding components in each group.

**Table 2:** Weighted principal components analysis (PCA) loadings for glutathione, glutamate, and glutamine in the whole sample. Bold font indicates loadings greater than 0.6.

3 principal components explaining 64.82% of the variance (varimax rotation)			
Variables	Component loadings		
	Component 1	Component 2	Component 3
Ins glutathione	**0.74**6	0.254	0.116
Ins glutamate	**0.808**	0.184	−0.059
Vis glutathione	**0.738**	0.028	0.013
Vis glutamate	**0.835**	0.070	−0.096
ACC glutathione	0.160	**0.871**	0.010
ACC glutamate	0.210	**0.804**	−0.080
ACC glutamine	−0.070	0.382	**0.725**
Ins glutamine	−0.004	−0.076	**0.759**
Vis glutamine	0.026	−0.168	**0.697**

There were no significant correlations between metabolite component scores and antipsychotic dose in either patient group, nor were there significant correlations with age. Significant positive correlations were observed between several of the metabolites, specifically between glutathione, glutamate, NAA, creatine in the ACC (ST6).

### Hierarchical models

**Fig. 3:**
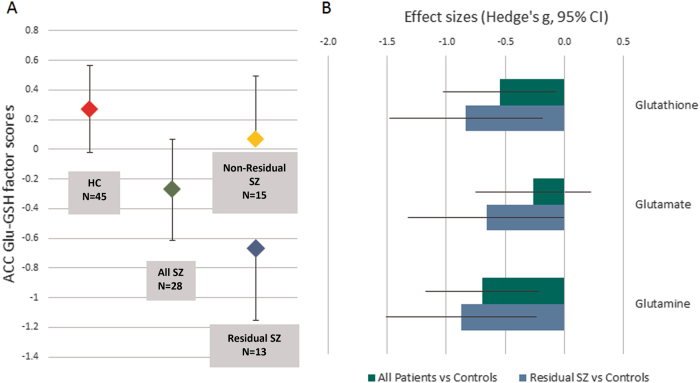
Panel **a** shows mean component scores for the second PCA component loading on glutathione and glutamate concentrations in the ACC. Error bars represent 95% confidence intervals. There were no significant differences in variances between any groups or sub-groups. Panel **b** shows the effect sizes for differences in mean metabolite concentrations in the ACC between healthy controls and all patients (green bars) and the sub-group of patients with residual schizophrenia (blue bars). Error bars represent 95% confidence limits of the effect size.

Our primary hypothesis concerned the ACC, glutathione, and glutamate. The second PCA component, loading on glutathione and glutamate scores in the ACC, was therefore our first dependent variable. When “Stable Schizophrenia” was entered as a predictor, the *R*^*2*^ of the model was 0.07, *F*(1, 71) = 5.332, *p* = 0.024, a small effect size, *f*^2^ = 0.08. The regression coefficient was significantly negative, indicating that mean component score was significantly lower in patients than in controls. When “Residual Schizophrenia” was included in the model, the *R*^*2*^ of the model increased to 0.12, a medium effect size, *f*^2^ = 0.14. This *R*^*2*^ increase was statistically significant, *F*(23, 70) = 4.114, *p* = 0.046. The regression coefficient for “Residual Schizophrenia” was significantly negative, indicating that mean score in patients with residual schizophrenia was significantly lower than in either the other patients or than healthy controls. In this model, “Stable Schizophrenia” was no longer a significant predictor, indicating that patient−control difference in the first model was being leveraged almost entirely by the presence in the patient group of the patients with residual schizophrenia. These results are shown graphically in Fig. 3a.

For the first and third PCA components (glutathione and glutamate in the Insula and Visual voxels; glutamine in all three voxels), neither predictor accounted for significant variance in component scores. Normality and homoscedasticity assumptions were satisfied in all models.

### Independent samples *t*-tests

Results from independent samples *t*-tests on the nine unadjusted metabolite concentrations (glutathione, glutamate, and glutamine in each of the three voxels) are given in Table 1. Group mean metabolite concentrations were significantly lower in the patients than in the controls for both glutathione and glutamine in the ACC, and significantly lower for all three metabolites in the ACC in the subset of patients with residual schizophrenia. Effect sizes are plotted in Fig. 3b.

## Discussion

Patients with stable schizophrenia had lower levels of glutathione and glutamate in the ACC, as measured by component scores representing variance shared between glutathione and glutamate in the ACC. This is consistent with our hypothesis that this group would include patients in whom excitotoxic damage during acute florid illness has led to subsequent reductions in mental and neural activity mediated by glutamatergic neurotransmission [3, 4]. Our hypothesis is further supported by the finding that for the sub-group of patients defined a priori as having residual schizophrenia, the effect size was larger, and indeed was largely responsible for the difference in scores between patients and controls. This finding is consistent with prior evidence indicating increased glutamatergic transmission early in the illness but diminished glutamate in older or more functionally impaired cases [29–37]. All three metabolites measured in the ACC were significantly lower in the group with residual schizophrenia than in the control group.

Glutathione and glutamate were significantly correlated with each other in all three brain regions. These high correlations are consistent with the hypothesis of a mechanistic link between antioxidant and glutamatergic systems in the human brain such that under steady-state conditions, low levels of glutathione are associated with low levels of glutamatergic neurotransmission [17]. In principle, the use of the unsuppressed water signal as a reference for metabolite signal normalization might introduce spurious correlations between glutathione and glutamate signals. However, provided variations in the water content within the sample are much smaller than the variations in the metabolite levels, variance shared between normalized metabolite signals can be assumed to arise largely from shared variance in metabolite concentrations [52]. It is noteworthy that there were no significant group differences in grey and white matter tissue composition in the three voxels (ST1).

Positive correlations exceeding an uncorrected threshold within each voxel were observed with all metabolites assessed apart for glutamine. After correction for multiple comparisons, correlations between glutathione, glutamate, NAA, creatine, and myo-inositol in the ACC remained significant. Component scores on a principal component representing variance shared between these five metabolites (ST7) were significantly reduced in residual schizophrenia compared with healthy controls. This suggests that these metabolites reflect metabolic integrity of cells (see SA8 for further discussion).

Ultra-high field MRS distinguishes between glutamate and glutamine, facilitating measures of glutathione and glutamate that are largely independent of glutamine levels. We found no significant correlations between glutamate and glutamine within any of the three voxels. However, we found significant correlations between glutamine levels across all three voxels, and all three glutamine measures loaded on a single component. This may be unsurprising, given the primary role of glutamine in glutamate recycling, a process likely to be cortex-wide.

Several limitations could be addressed in future studies. Firstly, given the evidence that glutathione and glutamate levels in the brain are affected by a risk variant of the gene coding for the glutathione synthesis enzyme, the role of genetic influences should be investigated [22]. Secondly, although we controlled for current antipsychotic medication intake and metabolite concentrations, possible confounding effects of long-term medication use should be considered. Thirdly, these results need to be interpreted with caution due to the potential differences in T1 and T2 relaxation times between different individuals and groups and their effects on neurochemical concentrations. Pragmatic constraints precluded measurement of water and metabolite T1 and T2 relaxation times in individual subjects. Instead, we used a short TE (17 ms) to minimize the effects of any potential differences in T2 relaxation times. While we cannot disregard the possibility of some remaining effects of relaxation time differentials, we note that our results are metabolite-specific, while systematic between-group differences in relaxation times would likely impact all metabolite concentrations equally.

Lastly, although the total number of patients was substantial, the cell size of the residual sub-group was small. Future studies with larger cell sizes should be conducted to compare sub-groups patients with different symptom profiles. Mostly importantly, our findings should motivate longitudinal studies using 7T MRS to follow neurochemical changes in individual patents across the phases of their illness.

## Disclaimer

None of the funding bodies played any role in design and conduct of the study; collection, management, analysis, and interpretation of the data; and preparation, review, or approval of the manuscript; and decision to submit the manuscript for publication.

## Electronic supplementary material

Supplementary Material
