# Supplementary material for: Glutathione and glutamate in schizophrenia: a 7T MRS study
Source: Mol Psychiatry. 2018 Jun 22;25(4):873–82. doi: 10.1038/s41380-018-0104-7 (PMC7156342; doi:10.1038/s41380-018-0104-7)
Supplement: Supplementary file 1 — Supplementary Material [file 41380_2018_104_MOESM1_ESM.docx]

# Supplementary Material

Appendices

**Methods**

SA1: Inclusion and exclusion criteria

SA2: Operational criteria for residual schizophrenia

SA3: Matching criteria

SA4: Details of assessments

SA5. Estimating metabolite concentrations

**Results**

SA6: Participant exclusions and sample size

SA7: Confounds and corrections

SA8: Correlations between glutathione, glutamate and other metabolites

**Tables**

ST1: Grey matter and white matter composition in all groups

ST2: MRS data quality measures in all groups

ST3: LCModel correlation co-efficiency (cc) values between metabolite fits for glutathione, glutamate and glutamine in all groups

ST4: Clinical and demographic features of the sample

ST5: Mean n-acetyl aspartate, creatine and myoinositol concentrations measured in millimolar (mM)) in the three voxels

ST6: Correlations between the metabolites in the ACC across the whole sample

ST7: Weighted Principal Components Analysis (PCA) loadings for all six metabolites (glutathione, glutamate, glutamine, n-acetyl aspartate, creatine and myoinositol) in the whole sample

**References**

**Methods**

SA1: Inclusion and exclusion criteria

Exclusion criteria were: 1) IQ below 70, 2) Lifetime history of substance dependence or harmful use in the past 6 months, 3) History of significant head trauma or medical conditions likely to have appreciable neurological or psychiatric effects 4) Contraindications for MRI safety assessed by a standardized safety screening questionnaire. A personal or family history of psychotic illness was an exclusion criterion for controls. Patients were included if: a) they satisfied DSM IV criteria for schizophrenia or schizoaffective disorder; this was determined by a consensus meeting in accordance with the best estimate procedure described by Leckman *et al*. (1982) (1) utilizing evidence regarding current clinical state and a retrospective review of case notes and b) if they satisfied the criteria for stable phase of illness, defined as a change of no more than 10 points in their Social and Occupational Functioning Assessment Scale (SOFAS) score (defined in DSM-IV (2)) between assessment 6 weeks prior to and immediately prior to study participation.

SA2: Operational criteria for residual schizophrenia

As the DSM does not include explicit criteria for residual schizophrenia, we created operational criteria reflecting the ICD-10 criteria. Our operational criteria were:

1. Cases of schizophrenia, currently in a stable phase of illness, defined by no change in SOFAS score of greater than 10 units in the preceding 6 weeks, who previously exhibited prominent delusions, hallucinations and or positive thought disorder, recorded in the case-file in an earlier phase of illness;
2. Currently exhibiting a combined score for delusions, hallucinations and positive thought disorder, assessed using the Signs and Symptoms of Psychotic Illness (SSPI) scale (3), no greater than 1 (a score of 1 indicates clinical features near the upper boundary of normal, and are questionably abnormal);
3. Currently exhibit a combined score for negative symptoms (poverty of speech, flat affect, decreased spontaneous movement and anhedonia) of 2 or greater, assessed using the SSPI scale (3) and/or a score for occupational and social function less than 70 assessed using the SOFAS scale (2).

It should be noted that the group of cases classified as non-residual cases in our study are expected to be heterogeneous, potentially including cases with substantial persisting positive symptoms, cases with no appreciable persisting negative symptoms and cases with no history of a florid episode of illness.

SA3: Matching criteria

The issue of appropriate matching to minimise risk of group differences in brain development unrelated to the pathophysiology of schizophrenia remains a subject of debate. There is consistent evidence that moderate lowering of IQ is related to the pathophysiology of schizophrenia and in particular, current IQ is influenced by factors such as age at onset of the disease, illness duration, severity of symptoms (4, 5). Therefore we did not match our groups for IQ. Nonetheless, to avoid inclusion of cases with IQ deficits greater than those typically observed in schizophrenia, we excluded individuals with a proxy estimate of current IQ lower than 70, as measured by the Quick Test (6). We instead used parental socio-economic status as a matching variable in this study. Parental occupational status might be expected to predict brain development (7, 8). Although there is evidence that parental occupational status is at least weakly predictive of risk of schizophrenia or of severity of illness, the evidence is ambiguous (9). We therefore consider that matching groups for parental occupational status is the most appropriate way to minimise risk of group differences in brain development that are not directly related to the pathophysiology of schizophrenia.

SA4: Details of assessments

On the day of scanning, handedness was assessed for both groups using the 12 items Annett scale (10). Social and occupational functioning was measured using the SOFAS scale (2). A video recorded clinical interview was also conducted by a trained team member using a standardized symptom assessment procedure (SSPI scale (3)). IQ was assessed using the Quick Test (6).

SA5: Estimating metabolite concentrations

The anatomical image was segmented into grey matter, white matter and cerebrospinal fluid (CSF) using SPM8 (11). Tissue volume fractions were calculated using the segmented images and spatial co-ordinates of voxels and are reported in table ST1. MRS data acquired independently from the 32 channels were reformatted, phase-corrected and realigned to account for frequency drifts, and then combined using an optimised coil combination method for MRS (12). Metabolite concentrations were estimated by fitting predicted spectra to the observed spectra using LCmodel (13) with a custom basis set which included 20 metabolites. Lipid resonances and macromolecules were also included during the LCModel fitting. Metabolite concentrations were normalised to the concentration of water calculated from the unsuppressed water spectra, as variations in the water content within the sample are expected to be much smaller than the variations in the metabolite level. The variance between subjects in water signal is typically in the range 1.5-2.5% (14) whereas variance in other metabolites that might be used as reference is typically around 5%. We assessed the reliability of the model fitting using Cramér-Rao Lower Bound (CRLB) estimates of the precision of the quantification of concentrations for each metabolite. Data above a CRLB threshold of SD=20% were excluded from the analysis (15). The calculated tissue fractions were used to correct metabolite concentrations for partial volume effects and relaxation attenuation in the voxel (16). T1 and T2 water relaxation times in grey matter, white matter and CSF were taken from previous published reports (17-19). MRS data quality measures in all groups are reported in ST2.

**Results**

SA6: Participant exclusions and sample size

A total of 46 healthy controls and 40 patients with schizophrenia and 1 patient with schizoaffective disorder were recruited for this study. 1 healthy control and 12 patients (including the one patient with schizoaffective disorder) did not undergo MRS data acquisition due to discomfort or anxiety arising from lying inside the scanner. We did not acquire MRS data from the visual voxel for 1 patient who partially completed MRS acquisition. Bad spectral quality and errors during data acquisition led to loss of ACC MRS data for one patient, insula and visual MRS data for another and data from all three voxels for a third patient. Thus, for all three voxels, data from 45 healthy controls were entered into subsequent analyses. In the patient group, data from 27, 27 and 26 patients were entered into subsequent analyses for the ACC, left insula and visual voxels respectively. ACC glutamine data for 3 healthy controls, insula and visual glutamine data for 1 healthy control and insula glutamine data for 1 patient had to be excluded as they did not meet the CRLB criteria.

As summarised in the Introduction to the main text, previous MRS studies at 3T or 4T had reported abnormalities in glutamate levels in medial frontal cortex/ACC in schizophrenia with medium effect size (Cohen’s d ~ 0.5). Power calculation using G-power (20) demonstrated that a sample of 64 cases of schizophrenia and 64 controls would be required to provide 80% power of detecting an abnormality of effect size d=0.5 at the alpha level p=0.05.

We anticipated greater power to detect abnormalities with MRS at 7T. Furthermore, the studies reviewed by Marsman *et al* (21) indicated heterogeneity, with glutamate increased relative to healthy controls in younger cases and decreased relative to controls in older cases. In light of the hypothesis that over-activity of glutamate at an early phase of illness might lead to cellular damage and decrease at later stages (22, 23) we predicted a reduction in glutamate levels of medium to large effect size in a subset of cases with residual illness.

Within the time period limited by available funding, we recruited 46 healthy controls and 41 patients with schizophrenia, of whom 15 satisfied our criteria for residual schizophrenia. Of these, adequate quality MRS data was acquired in 45 healthy participants and 28 cases of schizophrenia including 13 residual cases. Samples of these sizes provided 80% power to detect an effect of size d= 0.68 in the full sample of schizophrenia cases, and an effect of size d=0.9 in residual cases, at the alpha level p=0.5.

SA7: Confounds and corrections

There were no significant differences in grey and white matter tissue compositions in the three voxels between the groups (ST1). There was a significant difference in spectral line-width in the visual cortex between patients and controls (ST2). Hence, all visual cortex metabolites were regressed against visual line-width prior to further analyses. Since metabolite concentrations tended to be systematically higher in men than in women, metabolite concentrations were regressed against gender and the residuals i.e. gender-adjusted values of all metabolites were used in all analyses for all groups. We also inspected the correlation co-efficiency (cc) outputs from LCModel in order to ensure that any observed correlations between metabolite measures were not artefacts of LCModel fitting. The cc values for glutathione-glutamate, glutathione-glutamine and glutamate-glutamine in all three voxels in all groups were low and these have been reported in ST3.

SA8: Correlations between glutathione, glutamate and other metabolites

In the main text we hypothesized a relationship between glutamate and glutathione based on prior evidence from studies of animals and humans. Our finding supported this hypothesis. There are also grounds for anticipating relationships between glutamate, glutamine and/or glutathione and other metabolites, including creatine, N-acetyl aspartate (NAA) and myo-inositol, based on evidence regarding the physiological role of these metabolites and direct evidence from MRS studies in humans. Although detailed discussion of these relationships is beyond the scope of this manuscript, we provide a brief discussion here.

Creatine plays an important role in cellular energy metabolism. Creatine phosphate is a substrate for the synthesis of adenosine triphosphate (ATP) from adenosine diphosphate (ADP). NAA is commonly regarded as a marker to neuronal integrity and in particular can contribute to energy production from glutamate. Thus both creatine and NAA are markers of metabolically healthy cells. Similarly glutamate and glutamine, which are generated via a metabolic pathway branching off the tricarboxylic acid cycle central to oxidative energy metabolism, play important roles in cellular metabolic health. The relationship between glutamate and/or glutamine and energy metabolism would be expected to be of critical importance in the brain insofar as they are neurotransmitters and neurotransmission requires energy metabolism. Similarly, the role of glutathione as the major defence against oxidative stress would lead to the expectation of relationship between glutathione and metabolites related to oxidative metabolism.

Myo-inositol is commonly regarded as an osmolite, but is also the basic building block for phosphoinositides, which serve as second messengers mediating a wide range of cellular metabolic processes (24). The conversion of inositol monophosphate to myo-inositol plays an important part in the recycling of phosphoinositides to maintain phosphatidylinositol signalling. In depression, acute inflammation is associated with elevated glutamate and myo-inositol in basal ganglia (25). It is plausible that the cytotoxicity due to excess glutamatergic transmission and associated inflammation in the acute phase of schizophrenia, might result in diminution not only of glutamate but also of myo-inositol in the subsequent residual phase

Although evidence regarding observed relationships between these various metabolites in the human brain is sparse, the available evidence does indicate strong correlations. For example, Kaiser *et al* (26) reported correlations of *r* = 0.86 between glutamate and NAA; and *r* = 0.78 between glutamate and creatine.

The correlations between glutamate, glutamine, glutathione, creatine, NAAand myo-inositol in the ACC in the full sample in our study are reported in table ST6. The correlations between metabolites were generally of similar magnitude in all three voxels, apart for the observation that creatine exhibited only weak correlation with glutamate and glutathione in the insula (GSH – Cr: *r* = -0.02, *p* = 0.82, Glu – Cr: *r* = 0.06, *p* = 0.6) and with glutathione in visual cortex (GSH – Cr: *r* = 0.15, *p* = 0.20; Glu- Cr: *r* = 0.28, *p* = 0.01).

It is noteworthy that the cross-correlation values which reflect overlap between the models for pairs of metabolites employed in the fitting procedure in LC model (and indicate the risk of artefactual inflation of reported correlation between metabolite concentrations due to overlap of the models) were all less than 0.2 in absolute magnitude, apart from the cross-correlations between models for glutamate and NAA which had a mean value of 0.3. Thus, apart from a possible modest effect on correlations involving NAA, it is unlikely that overlap in the models caused appreciable inflation of the reported correlations between metabolite concentrations.

It should also be noted that correlations between metabolites might be inflated by the use of a shared reference, namely water. This is an issue whichever signal is used for reference, but as discussed by Neeb *et al.* (14), water is expected to produce less inflation of correlations than use of other metabolites as reference.

Thus our data reveal substantial correlation between pairs of metabolites, including correlations between glutamate and creatine, NAA, and myo-inositol and also between glutathione and creatine and NAA. Such correlations are consistent with expectation based on the physiological roles of these metabolites and published MRS findings (24).

In light of the evidence indicating substantial shared variance between multiple metabolites related to cellular metabolic integrity, we performed a Principal Component Analysis of these correlated metabolites together with glutamine, in the ACC. The first principal component exhibited heavy loadings on glutamate, glutathione, NAA, creatine and myo-inositol, but only a weak loading on glutamine, as shown in table ST7. This component might be described as a component reflecting ‘metabolic integrity’.

We then tested the hypothesis that scores on this metabolic integrity component are reduced in schizophrenia. As in the analysis reported in the main text, we performed a hierarchical regression analysis. The diagnosis of schizophrenia (v healthy controls) was entered as the predictor in the first block. This diagnosis of schizophrenia showed a trend toward being a significant predictor (*F*(1,71) = 3.242, *p* = 0.076) reflecting lower component scores in the patients. In the second block, diagnosis of residual schizophrenia was entered as an additional regressor. Adding the information about residual status increased the significance of the model (*F*(1,70) = 4.244, *p* = 0.018) indicating that the metabolic integrity factor was even more strongly reduced in residual cases. A t-test directly comparing the metabolic integrity score in patients with residual schizophrenia with that healthy controls confirmed the significant reduction in residual cases (t = 2.702, df = 56, *p* = 0.009).

Thus, it appears that the observed reduction in glutamate and glutathione in the ACC in stable schizophrenia, and especially in cases satisfying criteria for residual schizophrenia, is part of a more extensive reduction in a set of metabolites that might be regarded as indicator of metabolic integrity. This is indeed consistent with our underlying hypothesis that cytotoxic damage during the acute phase of illness leads to persisting metabolic damage that is especially pronounced in cases satisfying criteria for residual schizophrenia.

**Tables**

ST1: Grey matter and white matter volume fractions in all groups

|  |  | Tissue composition (Mean/SD) | | | Hedge’s g (LCL, UCL) |
| --- | --- | --- | --- | --- | --- |
| Region | Tissue Type | All  Patients | Residual Schizophrenia | Healthy Controls | All patients-HC |
| ACC | Grey matter | 0.729 (0.055) | 0.734 (0.058) | 0.743 (0.067) | -0.23 (-0.91,0.45) |
|  | White matter | 0.099 (0.044) | 0.108 (0.050) | 0.111 (0.064) | -0.23 (-0.9,0.45) |
| Vis | Grey matter | 0.627 (0.055) | 0.634 (0.061) | 0.637 (0.046) | -0.19 (-0.87,0.49) |
|  | White matter | 0.289 (0.056) | 0.289 (0.064) | 0.285 (0.051) | 0.07 (-0.61,0.76) |
| Ins | Grey matter | 0.663 (0.053) | 0.675 (0.042) | 0.676 (0.051) | -0.25 (-0.92,0.43) |
|  | White matter | 0.189 (0.079) | 0.190 (0.087) | 0.226 (0.085) | -0.45 (-1.13,0.23) |

ACC = anterior cingulate cortex, Vis = visual cortex, Ins = left insula

Group comparisons (All Patients vs Healthy Controls; Residual Schizophrenia vs Healthy Controls) showed no significant differences in tissue composition between the groups, Bonferroni corrected for 6 multiple comparisons, *α*= .008. Effect sizes for each tissue type in each voxel are shown above for the differences between All Patients and Healthy Controls (99.2% confidence intervals). We also checked these comparisons using two 3 x 2 x 2 ANOVAs: Voxel by Tissue-type by Group (either All Patients vs Healthy Controls or Residual Schizophrenia vs Healthy Controls). There were no significant effects of Group on either tissue type in any voxel nor across voxels. However, when comparing All Patients with Healthy Controls, there was a significant group by voxel interaction (collapsed across both tissue types) *F*(1,69)=14.755, p<.001). Follow-up tests on summed tissue type fractions showed that, as would be expected, patients showed significant reductions in total (WM+GM) tissue fraction in the two salience network voxels, namely, the ACC and insula, and that these reductions were significantly greater than the non-significant difference observed in the visual voxel. In the ACC, mean summed tissue fraction was 85% (SD=4%) in healthy controls and 83% (SD 4%) in patients, while in the insula, mean summed tissue fraction was 90% (SD=5%) in healthy controls and 85% (SD=7%) in patients.

ST2: MRS data quality measures in all groups

|  |  | (Mean/SD) | | |
| --- | --- | --- | --- | --- |
| Region | Spectral quality  measure | All  Patients | Residual Schizophrenia | Healthy Controls |
| ACC | Line-width (Hz) | 17.58 (4.01) | 17.18 (2.15) | 16.98 (4.20) |
|  | SNR | 32.40 (9.74) | 31.58 (9.42) | 33.76 (10.60) |
| Vis | Line-width (Hz)* | 15.16 (3.31) | 16.68 (4.27) | 13.11 (1.87) |
|  | SNR | 41.96 (10.09) | 42.18 (9.35) | 46.22 (8.21) |
| Ins | Line-width (Hz) | 16.33 (6.14) | 16.63 (4.77) | 14.61 (3.07) |
|  | SNR | 23.60 (10.98) | 25.00 (11.01) | 24.49 (8.00) |

ACC = anterior cingulate cortex, Vis = visual cortex, Ins = left insula

* = significant difference between healthy controls and all patients with stable schizophrenia and also between healthy controls and residual schizophrenia patients (*p*<.05)

Note: In some cases the data did not satisfy the assumptions of normality and homoscedasticity were not satisfied. Therefore, bootstrapped Bias-corrected accelerated 95% confidence intervals were computed (10,000 samples), and the p value remained significant.

ST3: LCModel correlation co-efficiency (cc) values between metabolite fits in all groups

|  | cc values: Mean (SD)) | | | Hedge’s g (LCL, UCL) |
| --- | --- | --- | --- | --- |
| Region  Metabolites | All  Patients | Residual Schizophrenia | Healthy Controls | All patients-HC |
| ACC GSH-Glu | 0.053 (0.096) | 0.064 (0.086) | 0.071 (0.112) | -0.17 (-0.84,0.51) |
| ACC GSH-Gln | -0.086 (0.125) | -0.069 (0.090) | -0.029 (0.080) | -0.56 (-1.26,0.14) |
| ACC Glu-Gln | -0.037 (0.045) | -0.046 (0.045) | -0.062 (0.050) | 0.51 (-0.19,1.2) |
| Vis GSH-Glu | -0.027 (0.087) | 0.018 (0.099) | -0.052 (0.051) | 0.37 (-0.32,1.06) |
| Vis GSH-Gln | -0.016 (0.070) | 0.001 (0.061) | -0.032 (0.078) | 0.21 (-0.48,0.9) |
| Vis Glu-Gln | -0.038 (0.046) | -0.054 (0.039) | -0.021 (0.044) | -0.37 (-1.07,0.32) |
| Ins GSH-Glu | 0.031 (0.129) | 0.041 (0.131) | -0.001 (0.110) | 0.27 (-0.41,0.95) |
| Ins GSH-Gln | -0.120 (0.057) | -0.103 (0.063) | -0.103 (0.078) | -0.23 (-0.92,0.45) |
| Ins Glu-Gln | -0.044 (0.047) | -0.044 (0.043) | -0.045 (0.045) | 0.02 (-0.67,0.71) |

ACC = anterior cingulate cortex; Vis = visual cortex; Ins = left insula; Gln = glutamine; Glu = glutamate, GSH = glutathione.

There were no significant differences in cc values between the groups (All Patients vs Healthy Controls; Residual Schizophrenia patients vs Healthy Controls, Bonferroni corrected for 9 multiple comparisons, *α*=.006). Effect sizes with 99.4% confidence intervals are shown for the differences between All Patients and Healthy Controls. We also checked for group effects using two 3 x 3 x 2 ANOVAs: Voxel by Metabolite pair by Group (All Patients vs Healthy Controls; Residual Schizophrenia patients vs Healthy Controls). There were no significant main effects of Group, nor significant interactions with Group.

ST4: Clinical and demographic features of the sample

| Features | All Patients  (N = 28) | Residual Schizophrenia  (N = 13) | Controls  (N = 45) |
| --- | --- | --- | --- |
|  | Mean/N (SD) | | |
| Gender (Male/Female) | 20/8 | 6/7 | 29/16 |
| Age | 27.18 (6.54) | 28.00 (6.46) | 27.89 (7.54) |
| Parental NS-SEC | 2.39 (1.72) | 1.92 (1.49) | 1.97 (1.43) |
| Mean Illness Duration (Months) | 54.42 (48.43) | 72.00 (56.02) | - |
| DDD Antipsychotics | 1.21 (0.69) | 1.37 (0.90) | - |
| DDD Mood Stabilizers | 0.05 (0.22) | 0.00 | - |
| DDD Antidepressants | 0.43 (0.91) | 0.39 (0.66) | - |
| SOFAS Score* | 57.35 (16.25) | 53.92 (17.00) | 88.73 (5.82) |
| Quick IQ* | 97.82 (9.66) | 100 (10.13) | 104 (9.69) |
| Anxiety/Depression* | 1.57 (1.23) | 1.31 (1.31) | 0.78 (0.85) |
| Reality Distortion* | 2.11 (2.40) | 0.38 (0.87) | 0.13 (0.54) |
| Psychomotor Poverty*^+^ | 2.29 (1.88) | 1.62 (1.44) | 0.24 (0.48) |
| Disorganization^+^ | 0.46 (0.63) | 0.62 (0.50) | 0.20 (0.54) |
| Psychomotor Excitation | 0.57 (1.03) | 0.62 (1.12) | 0.47 (0.84) |

NS-SEC: National Statistics Socio-Economic Classification; Symptom scores computed from SSPI: Signs and Symptoms of Psychotic Illness scale; DDD: Defined Daily Dose; SOFAS: Social and Occupational Functioning Assessment Scale.

* = significant difference between healthy controls and all patients with stable schizophrenia (*p* < 0.05)

+ = significant difference between healthy controls and residual schizophrenia patients (*p* < 0.05)

Note: In some cases the data did not satisfy the assumptions of normality and homoscedasticity were not satisfied. Therefore, bootstrapped Bias-corrected accelerated 95% confidence intervals were computed (10,000 samples), and the p values remained significant.

ST5: Mean n-acetyl aspartate, creatine and myoinositol concentrations measured in millimolar (mM)) in the three voxels, and effect sizes for group comparisons.

|  | Concentrations in millimolar (mean (SD)) | | | Hedge’s g (LCL, UCL) |
| --- | --- | --- | --- | --- |
| Region  Metabolites | All  Patients | Residual Schizophrenia | Healthy Controls | All patients-HC |
| ACC NAA | 5.11 (0.52) | 4.91 (0.54) | 5.15 (0.62) | -0.06 (-0.73,0.61) |
| ACC Cr | 2.90 (0.54) | 2.74 (0.49) | 2.98 (0.69) | -0.13 (-0.8,0.53) |
| ACC mI* | 3.47 (0.49) | 3.36 (0.52) | 3.68 (0.46) | -0.21 (-0.9,0.47) |
| Vis NAA | 6.03 (0.55) | 6.15 (0.62) | 6.13 (0.46) | 0.02 (-0.66,0.71) |
| Vis Cr | 2.61 (0.52) | 2.54 (0.57) | 2.60 (0.39) | -0.02 (-0.7,0.65) |
| Vis mI | 3.27 (0.50) | 3.06 (0.57) | 3.33 (0.39) | -0.02 (-0.71,0.66) |
| Ins NAA | 5.15 (0.65) | 5.36 (0.49) | 5.16 (0.51) | -0.43 (-1.1,0.25) |
| Ins Cr | 3.27 (0.50) | 3.28 (0.68) | 3.28 (0.57) | -0.15 (-0.83,0.53) |
| Ins mI | 3.68 (0.49) | 3.56 (0.53) | 3.87 (0.53) | -0.37 (-1.06,0.32) |

Cr = Creatine, NAA = N-acetyl Aspartate, mI = Myo-Inositol

There were no significant differences in metabolite concentrations between the groups (All Patients vs Healthy Controls; Residual Schizophrenia patients vs Healthy Controls, Bonferroni corrected for 9 multiple comparisons, *α*=.006). Effect sizes with 99.4% confidence intervals are shown for the differences between All Patients and Healthy Controls. We also checked for group effects using two 3 x 3 x 2 ANOVAs: Voxel by Metabolite by Group (All Patients vs Healthy Controls; Residual Schizophrenia patients vs Healthy Controls). There were no significant main effects of Group, nor significant interactions with Group.

ST6: Correlations between the metabolites in the ACC across the whole sample

| ACC: Glu |  |  |  |  |  |  |
| --- | --- | --- | --- | --- | --- | --- |
| ACC: Gln | 0.07 |  |  |  |  |  |
| ACC: GSH | 0.56* | 0.33 |  |  |  |  |
| ACC: Cr | 0.40* | 0.09 | 0.44* |  |  |  |
| ACC: NAA | 0.57* | -0.10 | 0.47* | 0.29 |  |  |
| ACC: mI | 0.48* | 0.10 | 0.30 | 0.31 | 0.23 |  |
|  | ACC: Glu | ACC: Gln | ACC: GSH | ACC: Cr | ACC: NAA | ACC: mI |

Glu = Glutamate, Gln = Glutamine, GSH = Glutathione, Cr = Creatine, NAA = N-acetyl Aspartate, mI = Myo-Inositol

* p<.05, Bonferroni corrected for 15 multiple comparisons

Max N pairwise = 73, Min N pairwise = 69

Note: As the data did not satisfy the assumption of multivariate normality for multiple pairwise correlations, bootstrapped Bias-corrected accelerated 95% confidence intervals were computed (10,000 samples), and the correlations remained significant.

ST7: Weighted Principal Components Analysis (PCA) loadings for all six metabolites (glutathione, glutamate, glutamine, n-acetyl aspartate, creatine and myoinositol) in the whole sample

| **2 principal components explaining 61.08% of the variance**  **(varimax rotation)** | | |
| --- | --- | --- |
|  | Component loadings | |
| Variables | Component 1 | Component 2 |
| ACC glutathione | **0.774** | 0.213 |
| ACC glutamate | **0.842** | -0.042 |
| ACC glutamine | 0.151 | **0.918** |
| ACC n-acetyl aspartate | **0.681** | -0.436 |
| ACC creatine | **0.624** | 0.056 |
| ACC myo-inositol | **0.608** | 0.165 |

**References**

1. Leckman JF, Sholomskas D, Thompson WD, Belanger A, Weissman MM. Best estimate of lifetime psychiatric diagnosis: a methodological study. *Arch Gen Psychiatry* 1982; **39(8)**: 879–83.
2. American Psychiatric Association: *Diagnostic and statistical manual of mental disorders: DSM-IV.* 1994; (4^th^ ed). Washington, DC, American Psychiatric Association.
3. Liddle PF, Ngan ETC, Duffield G, Kho K, Warren AJ. Signs and Symptoms of Psychotic Illness (SSPI): a rating scale. *Br J Psychiatry* 2002; **180**: 45–50.
4. Aylward E, Walker E, Bettes B. Intelligence in schizophrenia: meta-analysis of the research. *Schizophr Bull* 1984; **10(3)**: 430–59.
5. Woodberry KA, Giuliano AJ, Seidman LJ. Premorbid IQ in schizophrenia: a meta-analytic review. *Am J Psychiatry* 2008; **165(5)**: 579–87.
6. Ammons RB, Ammons CH. The Quick Test (QT): provisional manual. *Psychol Rep* 1962; **11(1)**: 111-61.
7. Noble KG, Norman MF, Farah MJ. Neurocognitive correlates of socioeconomic status in kindergarten children. *Dev Sci* 2005; **8(1)**: 74–87.
8. Farah MJ, Shera DM, Savage JH, Betancourt L, Giannetta JM, Brodsky NL et al. Childhood poverty: Specific associations with neurocognitive development. *Brain Res* 2006; **1110(1)**: 166–74.
9. Parrott B, Lewine R. Socioeconomic status of origin and the clinical expression of Schizophrenia. *Schizophr Res.* 2005; **75(2-3)**: 417–24.
10. Annett M. A classification of hand preference by association analysis. *Br J Psychol* 1970; **61(3)**: 303–21.
11. Ashburner J, Barnes G, Chen C, Daunizeau J, Flandin G, Friston K et al: *SPM8 manual*. London, Functional Imaging Laboratory, Institute of Neurology, 2012.
12. Hall EL, Stephenson MC, Price D, Morris PG: Methodology for improved detection of low concentration metabolites in MRS: optimised combination of signals from multi-element coil arrays. *Neuroimage* 2014; **86**: 35–42.
13. Provencher SW.Estimation of metabolite concentrations from localized in vivo proton NMR spectra. *Magn Reson Med* 1993; **30(6)**: 672–9.
14. Neeb H, Ermer V, Stocker T, Shah NJ. Fast quantitative mapping of absolute water content with full brain coverage*. Neuroimage* 2008; **42(3)**:1094-109.
15. Provencher SW. LCModel and LCMgui User’s Manual. http://www.s-provencher.com/pub/LCModel/manual/manual.pdf. Accessed July 1, 2015.
16. Gasparovic C, Song T, Devier D, Bockholt HJ, Caprihan A, Mullins PG et al. Use of tissue water as a concentration reference for proton spectroscopic imaging. *Magn Reson Med* 2006; **55(6)**: 1219–26.
17. Rooney WD, Johnson G, Li X, Cohen ER, Kim SG, Ugurbil K et al. Magnetic field and tissue dependencies of human brain longitudinal 1H2O relaxation in vivo. *Magn Reson Med* 2007; **57(2)**:308-18.
18. Cox EF, Gowland PA. Simultaneous quantification of T2 and T′ 2 using a combined gradient echo‐spin echo sequence at ultrahigh field*. Magn Reson Med* 2010; **64(5)**:1440-5.
19. Wright PJ, Mougin OE, Totman JJ, Peters AM, Brookes MJ, Coxon R et al. Water proton T1 measurements in brain tissue at 7, 3, and 1.5 T using IR-EPI, IR-TSE, and MPRAGE: results and optimization. *MAGMA* 2008; **21(1-2)**: 121-30.
20. Faul F, Erdfelder E, Lang AG, Buchner A. G* Power 3: A flexible statistical power analysis program for the social, behavioral, and biomedical sciences. *Behav Res Methods* 2007; **39(2)**: 175-91.
21. Marsman A, van den Heuvel MP, Klomp DW, Kahn RS, Luijten PR, Hulshoff Pol HE. Glutamate in schizophrenia: a focused review and meta-analysis of 1H-MRS studies. *Schizophr Bull* 2013; **39(1)**: 120–9.
22. Moghaddam B, Krystal JH. Capturing the angel in “angel dust”: twenty years of translational neuroscience studies of NMDA receptor antagonists in animals and humans. *Schizophr Bull* 2012; **38(5)**: 942-9.
23. Deutsch SI, Rosse RB, Schwartz BL, Mastropaolo J. A revised excitotoxic hypothesis of schizophrenia: therapeutic implications. *Clin Neuropharmacol* 2001; **24(1)**: 43-9.
24. Balla T. Phosphoinositides: tiny lipids with giant impact on cell regulation. *Physiol Rev* 2013; **93 (3)**: 1019-137.
25. Haroon E, Fleischer CC, Felger JC, Chen X, Woolwine BJ, Patel T et al. Conceptual convergence: increased inflammation is associated with increased basal ganglia glutamate in patients with major depression. *Molecular Psychiatry* 2016; **21**: 1351–1357.
26. Kaiser LG, Schuff N, Cashdollar N, Weiner MW. Age-related glutamate and glutamine concentration changes in normal human brain: 1 H MR spectroscopy study at 4 T. *Neurobiol Aging* 2005; **26(5)**: 665-72.
